# Antinociceptive and antiinflammatory activities of crude leave extract and solvent fractions of *Commelina latifolia* Hochst. ex C.B.Clarke (Commelinaceae) leaves in murine model

**DOI:** 10.3389/fphar.2023.1284087

**Published:** 2023-12-07

**Authors:** Getnet Tadege, Betelhem Sirak, Dehnnet Abebe, Dejen Nureye

**Affiliations:** ^1^ Department of Pharmacy, College of Health Sciences, Debre Markos University, Debre Marqos, Ethiopia; ^2^ Department of Pharmacy, College of Medicine Health Sciences, Arbaminch University, Arba Minch, Ethiopia; ^3^ School of Pharmacy, Institute of Health, Jimma University, Jimma, Oromia, Ethiopia; ^4^ School of Pharmacy, College of Medicine and Health Sciences, Mizan-Tepi University, Mizan-Aman, Ethiopia

**Keywords:** *Commelina*, traditional medicine, antinociception, antiinflammatory, carrageenan, writhing

## Abstract

**Ethnopharmacological relevance:** In the past, Ethiopian traditional medicine employed the leaves of the native *Commelina latifolia* Hochst. ex C.B. Clarke plant to treat wounds, pain, and malaria.

**Aim of the study:** The crude extract and solvent fractions of *C. latifolia* Hochst. ex C.B. Clarke leaves were examined in the present investigation to determine their ability to have an antiinflammatory effect and provide an antinociceptive effect in animal models.

**Materials and methods:** The leaves of *C. latifolia* were extracted with 80% methanol, and the CL crude extract was further fractionated with chloroform, pure methanol, and distilled water. The carrageenan-induced paw edema model was used to test the extracts’ ability to reduce inflammation. The hotplate model and the acetic acid-induced writhing test on rodents were used to test the extracts’ potential antinociceptive effect to reduce pain.

**Results:** Inflammation was decreased by 64.59% with CL crude extract (400 mg/kg); 56.34% (400 mg/kg) of methanol fraction, 64.59% of aqueous fraction (400 mg/kg), and 38.27% of chloroform fraction in the carrageenan-induced inflammatory model. All extracts demonstrated a considerable lengthening of the nociception reaction time in the hot plate test, with a maximum antinociceptive effect of 78.98% (crude extract) and 71.65% (solvent fractions). At a dosage of 400 mg/kg, the natural *C. latifolia* crude extract and aqueous fraction demonstrated considerable antinociceptive effects against acetylsalicylic acid (ASA) during the writhing test (48.83% and 45.37than%, respectively). The current findings support Ethiopia’s traditional user’s assertions that the herb can alleviate inflammation and pain.

## 1 Introduction

A physiological and psychological sensation known as pain is linked to actual or possible tissue injury This frequently serves as one of the disease’s early symptoms and indications and the primary reason patients visit their doctor (Raja et al., 2021). Additionally, the body’s most typical adaptive response is inflammation (Rochette et al., 2020). Both discomfort and inflammation include many kinds of complex biological reactions, such as the activation of enzymes, the release of inflammatory mediators and extravasating fluid, cell migration, and the breakdown and regeneration of tissue (Stolom et al., 2016).

Hence, using accessible antiinflammatory medications could potentially be beneficial in the prevention and management of inflammation (Tabas and Glass, 2013). Antiinflammatory medications, whether steroidal or non-steroidal, are frequently used to treat various inflammatory illnesses (Zheng and Du, 2014). Non-steroidal antiinflammatory drugs (NSAIDs) can cause stomach sores, while opiates can cause tolerance and dependency. Although these medications offer significant assistance in managing pain and inflammation, certain individuals may experience terrible side effects from them (Rochette et al., 2020). As a result, researchers worldwide are looking for novel NSAIDs and opiate replacements that do not have those side effects (Pergolizzi et al., 2021). Significant emphasis has been paid to examining the efficacy of herbal medicines used in conventional treatment. Since they are accessible, have few negative effects, and still account for about 80% of all drugs used by people globally, as reported by the World Health Organization (WHO) (Mahesh et al., 2021).

There are over 170 species in the genus *Commelina,* which is often known as dayflowers because of the brief lifespan of its flowers. They are frequently referred to as widow’s tears. It is by far the biggest genus in its family, Commelinaceae (Nampy et al., 2013). The genus is widely distributed all across the warm-temperate, subtropical, and tropical regions of Africa and Asia (Russell, 2015). It favors humid environments even though it is frequently found in woodlands and meadows (Russell, 2015). It is also naturalized in North and South America (Nampy et al., 2013; Russell, 2015). Species from this genus have been reported to possess medicinal properties for the management of pain and inflammatory disease. For example, the ethanol extract of *Commelina benghalensis* Linn. Possesses potential antinociceptive and antiinflammatory activities (Hossain et al., 2014). Ethiopians frequently utilize *Commelina latifolia* Hochst. ex C.B. Clarke (also known locally as Yewuha enqur or Yewof enqur) as a remedy for inflammation, wounds, and malaria (Abebe and Garedew, 2019). The fresh leave of *C. latifolia* crushed and the wound area is covered with crushed leave to prevent wound-induced pain and inflammation in different parts of Ethiopia. In addition to this, the fresh leave was chewed for the management of toothache (Chekole et al., 2015; Bitew et al., 2019). The whole part of the plant was medicinally used for various diseases traditionally. The herb is native to Ethiopia, Burundi, Central African Repu, Congo, Eritrea, Kenya, Rwanda, Somalia, Sudan, Tanzania, Uganda, and Yemen, Zaïre (Faden, 1994; Patil et al., 2019). *Commelina latifolia* is an herbaceous, perennial plant growing about 45 cm tall. Even though, the *in vivo* antiplasmodial effect of *C. latifolia* leave extracts (80% methanol, chloroform, methanol, and aqueous fractions) were reported previously (Tadege et al., 2022), the potential suppression of inflammation and pain relief effect was not confirmed yet. Therefore, the primary goal of this work was to evaluate the antinociceptive and antiinflammatory properties of the CL crude extract and solvent fractions of *C. latifolia*.

## 2 Materials and methods

### 2.1 Chemicals and drugs

The chemicals and drugs utilized in this experiment included carrageenan (Sigma Aldrich, Germany), water that had been distilled (EPHARM), analytical grade methanol (Carlo-Erba, France), 0.6% acetic acid (Sigma-Aldrich, USA), acetylsalicylic acid (ASA Cardio, Germany), indomethacin (Indocin^®^, Germany) and morphine (Sandoz, Germany) as a standard drug.

### 2.2 Materials

A conical flask, cylinder, test tubes, filtering funnel, filtering flask, filter paper, oral gavage, syringes, scissors, and permanent marker were among the supplies used in this experiment.

### 2.3 Equipment

Hot plates, electronic balance, rotary evaporator (Buchi, Switzerland), lyophilizer (Operon, Korean), and digital plethysmometer, were the tools and supplies used to conduct the experiments.

### 2.4 Collection and study plant Authenticaltion

The newly harvested leaves of *C. latifolia* were taken (on 23 September 2022, at 9:00 p.m.)with permission from the local community leaders in the Tepi Town of Ethiopia, about 611 km southwest of Addis Ababa. Voucher specimens were verified and placed in the National Herbarium, College of Natural and Computational Sciences, Addis Abeba University (DN003).

### 2.5 Extraction and fractionation

The leaves were manually cut into little pieces after being properly cleaned with distilled water to remove dirt. After that, they were dried in a well-ventilated area under cover after being crushed with the aid of a pestle and mortar into a finely ground substance for extraction. Then, each 500 g piece of dried, ground-up plant material was soaked for 72 hours in methanol with a concentration of 80% while being manually shaken often. Filters for mixtures included gauze and Whatman filter paper number 1, which has pores of 150 mm diameter. The methanol in the extract’s filtrate was evaporated using a rotary evaporator at 45 rpm and 40°C under reduced pressure to produce an extract with an 80% methanol concentration. Utilizing a lyophilizer and vacuum pressure (200 mBar), the concentrate was then subsequently dried. The yield percentage for the dry leaf CL extract, measured as net weight, was 64.28 g (12.86%). The concentrated CL extract was stored at −4°C in the freezer until use. Using a freeze-drying device, the CL extract being extracted was subsequently dehydrated. After being placed into vials, the 64.28 g dry extract (13.71% w/w) was maintained at −20°C until use. Using a Soxhlet extractor, the concentrated hydroalcoholic CL crude extract (44.75 g) was afterward sequentially extracted with chloroform, 100% methanol, and distilled water. The solvents that were utilized in the methanol fraction (CL-MF) and chloroform fraction (CL-CF) were evaporated using a rotating evaporator, and the aqueous fraction (CL-AF) was dried and concentrated using a lyophilizer. 10.89 g (24.34%), 13.24 g (29.58%), and 18.34 g (40.98%) were the computed yields in percentages for the dried chloroform, methanol, and water phases. The fractions were kept at −20°C until they were needed.

### 2.6 Experimental animals

To test the hot plate and acetic acid-triggered writhing methods, the pharmacy at Mizan-Tepi University provided 240 normal Swiss albino mice of either sex, six to 8 weeks old, and weighing between 24 and 32 g. To determine the extracts’ ability to reduce inflammation brought on by carrageenan, the Ethiopian Public Health Institute (EPHI) supplied 120 Wistar albino rats with 250–350 g, 4–6 month old. For acclimatization, animals were kept in standard living conditions. They were fed pellets regularly and had unrestricted access to water while housed in a metal enclosure with a cycle of 12 h of light and darkness. The study’s techniques and processes were all carried out by the manual for the care and use of laboratory animals (NIH, 1996). The experimental method was approved by the Mizan Tepi University College of Medicine and Health Sciences, School of Pharmacy’s Ethical Review Committee with the protocol number of the ethics committee HSC/00445/2023.

### 2.7 Experimental models

#### 2.7.1 Carrageenan induced paw edema

Based on preventing the development of carrageenan-induced rat’s hind paw edema as reported previously by (Yonathan et al., 2006), *in vivo* antiinflammatory efficiency was assessed. Rats were fasted for 12 h before experimenting while having unlimited access to water. In this experiment, five groups of rats of both sexes, each group consisting of 6 rats, were employed (an equal number of both sexes). As vehicle (VEH) and positive controls, the first two groups received the VEH and 10 mg/kg indomethacin, respectively, throughout the tests. The rest of the groups were given extract doses of 100, 200, and 400 mg/kg. Every treatment was administered orally via oral gavage. A single dose of 0.05 mL of 1% carrageenan diluted regular saline was injected into the sub-plantar surface of the left hind paw 30 min after oral administration of the experimental extracts, the standard, and the VEH. Using a Ugo-Basile plethysmometer, the volumetric measurements of the injected paws were determined one, two, three, and 5 h after the injection of carrageenan. Oral administration of the entire dosage was limited to 10 mL/kg. Both the extract, CL-AF, and the indomethacin were dissolved in distilled water, and CL-MF and CL-CF were dissolved in 80% Tween 80 to make an oral solution.

The corresponding rise in paw volume, or swelling, was calculated in terms of percentages using the formula stated in (Delporte et al., 2005) as follows:

$$\%I=\frac{Vf-Vi}{Vi}\times 100$$ (1)

Where Vi denotes the volume of the paw before the carrageenan injection and Vf denotes the volume of the paw at a certain time following the injection. Additionally, the percentage-based antiinflammatory effect was calculated using the formula shown in Asefa et al. (2022):

$$\%A=\frac{\%Ic-\%Ie}{\%Ic}\times 100$$ (2)

The average levels of inflammation that were reached in the control and experimental groups, respectively, are represented by the numbers %Ic and %Ie.

#### 2.7.2 Hot plate test

This test was used to assess the antinociceptivec activity of herbal extracts by exposing animals to thermal discomfort stimuli and calculating the duration it took for a pain reaction to arise as the threshold for acute pain. Five groups of six mice each, representing both sexes, were established. Overnight, all animals were fasted. A positive control group of 10 mg/kg oral morphine was administered to one group, and three groups received varying doses of the plant extract (based on acute toxicity, p.o.). Another group received a VEH (control, p.o.). Each mouse was placed on a heated plate that was kept at 55°C (Asefa, Teshome, and Degu, 2022). Each mouse that was put through the test had its pain response time, or latency per second, assessed between the moment the animal was placed on a hot plate and when it began to kick, jump, lick, or grip its hind limbs. The time limit was set at 15 s to prevent any heat harm to the paw. Pain reaction times were assessed before and at 30, 60, 90, and 120 min after treatment to assess the antinociceptive effect of extract at various doses and the time effect response. Comparisons were made between the lengthening of delay times in groups receiving treatment and the value in the VEH groups. Using this method, it was possible to determine the proportion of antinociceptive maximal effect by using the following formula (Sedighi et al., 2014).

$$\text{maximum}antinociception=\frac{A-B}{15\;\mathrm{Sec}-B}\times 100$$ (3)

➢ Where A represents, the reaction time for test substance-administered mice, and B represents, the reaction time for VEH-administered mice

#### 2.7.3 Acetic acid-induced writhing test

This test was carried out as previously reported in Dash et al. (2015). There were five groups of six mice each, including mice of either sex in each group. A VEH was given to the control group, unlike the reference group, which received 0.6% acetic acid (10 mL/kg, i.p.) just 1 h after receiving 150 mg/kg of ASA. The plant extract was given in three groups at various doses (Shreedhara et al., 2009). To evaluate the antinociceptive activity of the CL crude extract and solvent fractions, the number of writhes was counted 5 min following an intraperitoneal injection of acetic acid. Each animal was placed in a glass container for 30 min while its abdominal muscles were collectively counted along with the stretching of its rear limbs. The study’s doses of the test chemicals were chosen by the plant’s previously stated safety profile (Tadege et al., 2022).

The percentage of protection against writhing was employed (Le Bars et al., 2001; Shreedhara et al., 2009) as an indicator of antinociception and was computed using the formula shown below:

$$\%\;\text{antinociceptive activity}=\frac{\text{MWC}-\text{MWT}}{\text{MWC}}\times 100$$ (4)

➢ Mean writhing counts for control groups and test groups are denoted by MWC and MWT, respectively.

### 2.8 Quality control

All of the chemicals, reagents, and instruments used were of analytical quality. The quality of the data was controlled by the pre-test (pilot study), acclimation, strict protocol adherence, randomization in the experimental mouse grouping, and coding microscopic slides in the blood smear production procedure. Furthermore, the impact of outside stimuli was lessened when naive mice were employed and kept in traditional laboratory conditions. To keep the cages hygienic, the animal attendants cleaned and took out the trash every other day. Medical lab employees counted erythrocytes without parasitization and those that were infected blindly.

### 2.9 Data analysis

The information was recorded into a worksheet in Microsoft Excel 2019 and then imported to SPSS version 26 for analysis. The mean ± standard error of the mean (SEM) of six animals were used per group. The standard deviation (SD = 10), which is a representation of the variability that we expect to find between subjects with the same treatment, was taken into consideration when calculating the sample size for each group. This was done by taking into account the average values of the various experimental groups, which refers to the estimate of the value that could represent a minimal significant relief with clinical relevance (20% antinociceptive and antiinflammatory effects) in comparison with a lack of relief of 5% (µ2-µ1 = 15); furthermore, a statistical power of 80% (Zβ = 0.84) was considered when calculating the sample size for each group. *p* < 0.05 indicates 5% of the likelihood that the observed difference is the result of chance, while the test’s power (80%) indicates the likelihood of detecting a statistically significant difference. Using these parameters and the formula (Charan and Kantharia, 2013; Kang, 2021; Ventura-Martinez et al., 2021), a n = 6.96 was computed. In order to reduce needless resource waste and ethical concerns, we employed six animals for this purpose, based on antinociceptive and anti-inflammatory animal models that have been tested in the past and show no significant differences from the computed value (Charan and Kantharia, 2013). Therefore, using six mice per group allows for sufficient statistical power while minimizing the number of animals needed for the study (Serdar et al., 2021).

$$n=\frac{2\times {SD}^{2}{\left(Z\alpha /2+Z\beta \right)}^{2}}{{\left(µ2-µ1\right)}^{2}}$$ (5)

The antinociceptive and antiinflammatory effects of CL extract and solvent fractions of C. latifolia were compared to the effects of VEH using a one-way analysis of variance (ANOVA) and a Dunnett’s *post hoc* test. Statistics were considered significant at a *p*-value less than 0.05. Graphs were created with the GraphPad Prism version 8.0.2 software.

## 3 Results

### 3.1 Carrageenan-inducednduced paw edema

#### 3.1.1 CL crude extract

**FIGURE 1:**
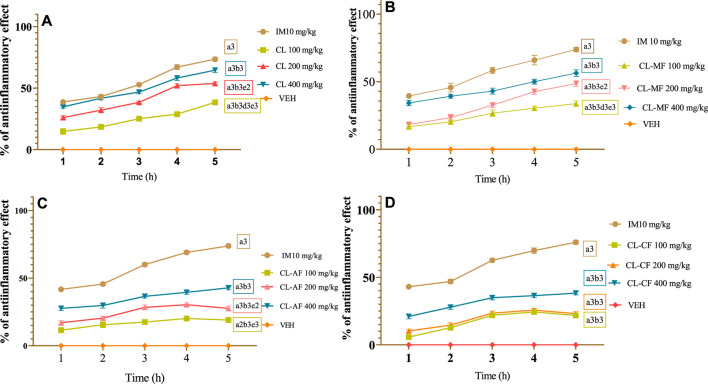
antiinflammatory effect of crude extract and solvent fraction of *Commelina latifolia* in carrageenan-induced paw edema at doses of 100, 200, and 400 mg/kg. **(A)** Antiinflammatory effect of CL crude extract. **(B)** Antiinflammatory effect of methanol fraction. **(C)** Antiinflammatory effect of aqueous fraction. **(D)** Antiinflammatory effect of chloroform fraction. CL, *Commelina latifolia*; CL-MF, methanol fraction of *Commelina latifolia*; CL-AF, aqueous fraction of *Commelina latifolia*; CLCF, chloroform fraction of *Commelina latifolia*; IM, indomethacin 10 mg/kg; and VEH, vehicle for reconstitution of extracts and drugs. Analysis was performed with one-way ANOVA followed by Tukey *post hoc* test. Data were expressed in mean ± SEM. n = 6; compared to a, VEH; b, IM 10 mg/kg; c, 100 mg/kg; d, 200 mg/kg; e, 400 mg/kg; 1, *p* < 0.05; 2, *p* < 0.01; 3, *p* < 0.001.

From the first to the fifth hours following carrageenan-induced paw edema, the CL crude extract at the doses of 200 and 400 mg/kg showed a significant (*p* < 0.001) suppression of paw edema in contrast to control groups. The strongest antiinflammatory effect of CL crude extract was seen in all three doses 5 h after the onset of edema, with dose-dependent inhibitions of 38.4%, 53.81%, and 64.59%, respectively (Figure 1A). While the indomethacin resulted in 73.53% at a dose of 10 mg/kg. Despite having less of an antiinflammatory effect than indomethacin (10 mg/kg), an intergroup comparison showed that CL200 and CL400 had comparable antiinflammatory effects throughout the observation period. It became noticeable over time that the extracts had antiinflammatory properties, notably for CL200 and CL400; this could indicate that a dose greater than 200 mg/kg is necessary to cause an antiinflammatory response. After 3 h, both the extracts and the usual medication showed significant antiinflammatory efficiency: this might be attributed to the time it takes for both substances to travel to the site of action.

#### 3.1.2 Solvent fractions

This experiment was conducted to determine the extracts’ ability to reduce inflammation after carrageenan injections to induce inflammatory pain. Beginning the second hour following the carrageenan injection, all three doses of CL-MF (100, 200, and 400 mg/kg) showed a significant suppression (*p* < 0.001) of the paw edema in comparison to the VEH group (Figure 1B). However, only the higher concentrations of CL-AF and CL-CF significantly reduced the edema induced by the sub-plantar injection of carrageenan (*p* < 0.001 at 2 h). 400 mg/kg doses of CL-MF (Figure 1B) provided the greatest protection against paw volume increases, with suppression values of 56.34% at the fifth hour, while CL-AF (Figure 1C) and CL-CF (Figure 1D) showed their maximum protection at the fourth hour with suppression values of 42.79%, and 38.27%, respectively. While the group received indomethacin had a 75.91% protective effect. Carrageenan was injected sub-plantar in groups that had been given solvent treatment, resulting in the formation of edema that progressively increased with time. Oral administration of CL-MF at doses of 100, 200, and 400 mg/kg maximally reduced the edema with respective inhibition levels of 33.76%, 48.61%, and 56.34% at the end of the fifth hour (Figure 1B).

### 3.2 Antinociceptiveactivities

#### 3.2.1 Hot plate test

##### 3.2.1.1 CL crude extract

**FIGURE 2:**
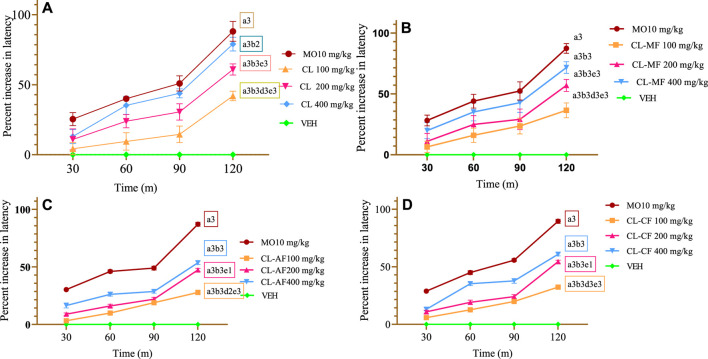
Antinociceptive effect of crude extract and solvent fraction of *Commelina latifolia* in hot plate teste at doses of 100, 200, and 400 mg/kg. **(A)** Antinociceptive effect of CL crude extract. **(B)** Antinociceptive effect of methanol fraction. **(C)** Antinociceptive effect of aqueous fraction. **(D)** Antinociceptive effect of chloroform fraction. CL, *Commelina latifolia*; CL-MF, methanol fraction of *Commelina latifolia*; CL-AF, aqueous fraction of *Commelina latifolia*; CL-CF, chloroform fraction of *Commelina latifolia*; MO, morphine 10 mg/kg; and VEH, vehicle for reconstitution of extracts and drugs. Analysis was performed with one-way ANOVA followed by Tukey *post hoc* test. Data were expressed in mean ± SEM. n = 6; compared to a, VEH; B, MO 10 mg/kg; c, 100 mg/kg; d, 200 mg/kg; e, 400 mg/kg; 1, *p* < 0.05; 2, *p* < 0.01; 3, *p* < 0.001.

The CL crude extract showed a dose-dependent increase in latency time at all three provided doses as compared to the VEH group that received VEH. An early, substantial (*p* < 0.05 at 30 min) antinociceptive impact was seen in both the CL200 and CL400 groups. The percentage inhibition of CL100, CL200, CL400, and morphine (MO10), respectively, was 41.96%, 60.87%, 78.98%, and 88.09% at 120 min (*p* < 0.001) (Figure 2A). Throughout the experiment, CL400 surpassed all extract groups in terms of % protection at a significant level (*p* < 0.001). Additionally, during the observation, the mean difference between the intermediate dosage and lower dose levels was substantial (*p* < 0.001 at 60, 90, and 120 min and *p* < 0.05 at 30 min). When compared to MO10, CL400 was shown to have a lower percentage of inhibition (*p* < 0.001 at 120 min and 90 min), nevertheless. The MO10 (positive control) group had a significantly delayed pain reaction time (*p* < 0.001) than the test substances and control (Figure 2A).

##### 3.2.1.2 Solvent fractions

With protection of 71.65% at a dose of 400 mg/kg (Figure 2B), the CL-MF showed the largest antinociception effect, followed by the CL-CF (60.86%) (Figure 2D) and the CL-AF (53.62%) (Figure 2C) after 120 min of observation in the hot plate test. Even though the CL-MF resulted the greatest latency period, it is a delayed nociception reaction less comparable with the CL crude extract. None of the CL-AF, CL-MF, or CL-CF, except 400 mg/kg of the CL-AF and CL-MF, significantly slowed the nociception reaction at 30 min as compared to the VEH group (*p* < 0.05). This demonstrates that the effects associated with usage were significant when measured across time frames greater than 30 min.

All three doses of solvent fractions started a significant protection effect at 60 min of observation, with equivalent protection observed on the chloroform (35.24%) and methanol (35.14%) fractions significantly (*p* < 0.001). In addition to this, the maximum protective effect was observed at 120 min of evaluation for all three fractions with their respective three doses. The average protection difference between 200 mg/kg and 100 mg/kg was observed at 120 min of evaluation (*p* < 0.001 for chloroform fraction, *p* < 0.01 for aqueous fraction, and *p* < 0.05 for methanol fraction) (Figure 2). The MO10 group had a significantly delayed pain reaction time (*p* < 0.001) than test substances and control at all observation times.

#### 3.2.2 Acetic acid-induced writhing method

##### 3.2.2.1 CL crude extract

**FIGURE 3:**
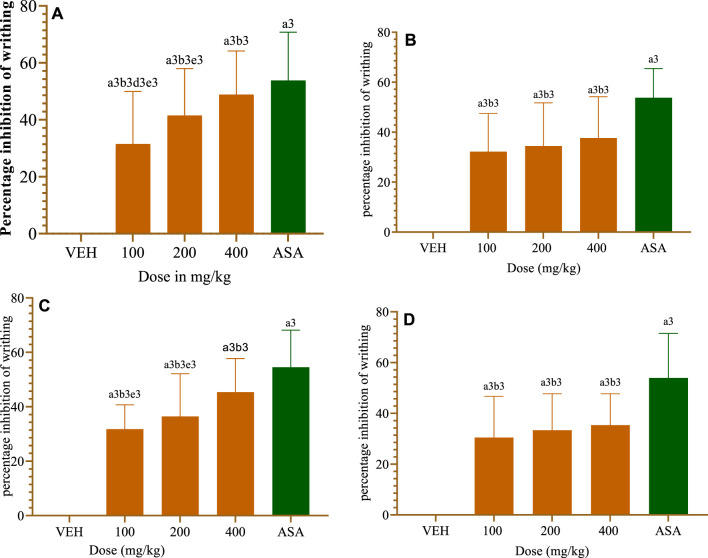
Antinociceptive effect of crude extract and a solvent fraction of *Commelina latifolia* in acetic acid induced writhing teste at doses of 100, 200 and 400 mg/kg. **(A)** Antinociceptive effect of CL crude extract. **(B)** Antinociceptive effect of methanol fraction. **(C)** Antinociceptive effect of aqueous fraction. **(D)** Antinociceptive effect of chloroform fraction. CL, *Commelina latifolia*; CL-MF, methanol fraction of *Commelina latifolia*; CL-AF, aqueous fraction of *Commelina latifolia*; CL-CF, chloroform fraction of *Commelina latifolia*; MO, morphine 10 mg/kg; and VEH, vehicle for reconstitution of extracts and drugs. Analysis was performed with one-way ANOVA followed by Tukey *post hoc* test. Data were expressed in mean ± SEM. n = 6; compared to a, VEH; b, IM 10 mg/kg; c, 100 mg/kg; d, 200 mg/kg; e, 400 mg/kg; 1, *p* < 0.05; 2, *p* < 0.01; 3, *p* < 0.001.

All test doses of the crude extract considerably (*p* < 0.001) reduced the acetic acid-induced writhing in mice as compared to the VEH group (Figure 3A). When compared to lower dosages of the plant extract, the 400 mg/kg dose of CL (CL400) showed an antinociceptive effect against the acetic acid-induced writhing response in mice (*p* < 0.001). ASA (150 mg/kg) similarly demonstrated a strong antinociceptive response compared to the extract doses of 100 mg/kg and 200 mg/kg (*p* < 0.001). However, the CL400 has an equivalent antinociceptive effect to the conventional drug, ASA 150 mg/kg.

##### 3.2.2.2 Solvent fractions

All test doses of solvent fractions drastically (*p* < 0.001) reduced the acetic acid-induced writhing in mice relative to the VEH-administered groups (Figure 3). Only the CL-AF of the three fractions significantly (*p* < 0.001) and gradually reduced the writhing that mice experience when exposed to acetic acid (Figure 3C). The 400 mg/kg dose of CL-AF had the strongest (45.37%) antinociception efficacy against the acetic acid-induced writhing response in mice (*p* < 0.01) in comparison to the 100 and 200 mg/kg doses of the CL-AF extracts. CL-MF (Figure 3B) and CL-CF(Figure 3D) had a significant antinociceptive effect in comparison to VEH received groups.

## 4 Discussion

In Ethiopia, a folklore herbal remedy called *C. latifolia* has been utilized to relieve pain and inflammation (Abebe and Garedew, 2019). However, science has not yet validated its medicinal antinociceptive and antiinflammatory capabilities. The goal of the current investigation was to determine whether CL crude extract and its solvent fractions had any antinociceptive or antiinflammatory activities. The carrageenan-induced rat paw edema model was used to evaluate the extracts’ anti-inflammatory properties, while the acetic acid-induced writhing mouse model and hot plate paradigm were used to analyze their antinociceptive properties.

Research for new antiinflammatory drugs frequently uses rat paw edema caused by carrageenan as a working model of inflammation (Ratheesh and Helen, 2007; Patil et al., 2019). CL reversed the inflammation and edema that the substance’s carrageenan-triggered model had caused (Ahmad et al., 2015; Man et al., 2019). This experimental paradigm was utilized to evaluate how the solvent and CL crude extract fractions affected acute inflammation (Esra et al., 2007). Because it does not have any systemic effects and does not cause any antigenic reactions, carrageenan is a favored testing agent for antiinflammatory medications (Akah and Nwambie, 1994; Jia et al., 2003).

There is uncertainty regarding the precise mechanism by which carrageenan causes inflammation in experimental animal models, but it has been shown to reduce the amount of epithelial glycoproteins in the paw (Al-Suhail et al., 1984) and to block the interaction of lymphocytes and macrophages (Chong and Parish, 1985). When carrageenan was used as a pretreatment, the severity of paw inflammation was raised (both clinically and histologically), and IL-6, TNF-α, and IL-10 expression were also increased (Wei et al., 2016). It involves the biphasic in releasing various chemical inflammatory agents, such as bradykinin, histamines, serotonin, and prostaglandins, which cause discomfort and fever (Ghosh et al., 2019; Uzor, 2020). This theory explains why the majority of animal investigations have concluded that carrageenan causes inflammation. Therefore, interferences with these processes, especially in later stages, could be a part of CL’s antiinflammatory action. These findings demonstrate that CL significantly affects granuloma formation, demonstrating its potency against chronic inflammation. A unique kind of persistent inflammation known as the granulomatous inflammatory response is typified by localized clusters of macrophages, epithelioid cells, and multinucleated giant cells. A granuloma is a focal aggregate of immune cells that forms in response to a persistent inflammatory stimulus. It characteristically demonstrates the compact organization of mature macrophages, which may or may not be associated with other inflammatory cell types (Magnani and Mahlaoui, 2016). The primary chemical components of plants that have analgesic and antiinflammatory activities are flavonoids, alkaloids, saponins, tannins, phenolic substances, glycosides, coumarins, and triterpenoids (Hussein and El-Anssary, 2019).

Alkaloids, cardiac glycosides, flavonoids, phenolic compounds, phlobatannins, tannins, and terpenoids were secondary metabolites identified by the preliminary phytochemical screening, as reported by Tadege et al., 2022 (17), in the CL crude extract. Because of the synergistic effects of various chemical components, the highest antiinflammatory effect seen in the crude extract of C. latifolia may have been caused by this (Panda et al., 2016). Due to the capability of suppressing enzymes involved in inflammation, particularly the arachidonic acid metabolic pathway and prostaglandin formation, tannins, flavonoids, and saponins are well known for their ability to decrease pain and have antiinflammatory characteristics (Sengupta and Sheorey, 2012). Tannins may influence the inflammatory reaction by inhibiting iNOS in macrophages and scavenging free radicals (Wang et al., 2019). On the other side, saponins lessen discomfort and inflammation by suppressing nitric oxide release (Suresh et al., 2021; Tan et al., 2022). Therefore, saponins, alkaloids, flavonoids, tannins, terpenoids, and phenols may be responsible for CL’s effects on pain relief and inflammation.

The dose-related delay time for the plant extracts and morphine (10 mg/kg) in the hot plate evaluation was also longer than for the control group. It is believed that the hot plate method is appropriate for drugs with a central effect (Koivisto et al., 2022). Because it evaluates the complex sensitivity to a non-inflammatory, acute nociceptive stimulus, the hot plate test is one of the common models used to study central nociceptive activity (Peerzada et al., 2020). This approach is well known that any substance that increases the delay time using this test must work primarily in the central nervous system (Ullah et al., 2014; Patil et al., 2019). As a result, the plant’s crude extract and solvent fractions must possess a core activity. Once more, NSAIDs only block peripheral pain, whereas opiates antinociceptive block both peripheral and central mechanisms of pain (Dévora et al., 2015; Silva-correa et al., 2021). Both forms of pain inhibition were observed in the CL crude extracts from both hydroalcoholic and solvent fractions (Sarmento-Neto et al., 2016).

The CL was successful in reducing acetic acid-related discomfort. The effectiveness of test compounds as peripheral antinociceptive is determined using the acetic acid-induced writhing in mice paradigm. According to this hypothesis, activation of the nerve terminal’s nociceptive fibers causes capillary permeability, which in turn lowers the nociceptive threshold, to generate inflammation-related pain (Walters, 2018; Dusi, 2020). The chemical acetic acid increased PGE2 and PGF2 release at the peritoneal receptors may potentially cause pain (Muhammad, 2014; Singh et al., 2016). Thus, the antinociceptive effect of CL may be due to interference of these nociceptive targets. In this investigation, the protective effect of acetic acid-induced writhing pain was diminished in all CL crude extract and solvent fractions. Thus, *C. latifolia* has a potential activity of central nociceptive activity than peripheral.

The findings of this investigation indicate that crude hydro-methanolic extract and solvent fractions of C. latifolia have antinociceptive effects on pain models produced by thermal and chemical noxious stimuli as well as antiinflammatory effects on paw edema brought on by carrageenan. The study endorsed the use of *C. latifolia* leaves locally for the management of inflammatory and pain problems.

This study employed the conventional models to analyze antiinflammatory and antinociceptive impact of the CL crude extract and solvent fractions of *C. latifolia* leaves was deemed the strength of the study. Restrictions on the research financial constraints prevented the isolation and characterization of the main constituents of the most active fractionate that gave rise to the plant’s antinociceptive and antiinflammatory properties, which could have served as a lead compound or compounds in the discovery and development of potent medications for the management of pain and inflammation.

## 5 Conclusion

The results of the current study confirmed that *C. latifolia* had promising antiinflammatory and antinociceptive activity in animal models. The different levels of protection from edema caused by carrageenan also contributed to the formation of the solvent fractions, with the methanol fraction being substantially more successful. Also, the plant showed potential antinociceptive activity against heat and acetic acid-induced pain. Our findings support Ethiopian traditional users’ claims that the plant can alleviate pain and inflammation. The active components that account for the antiinflammatory and antinociceptive properties of C. latifolia leaves must be found and isolated in the future.

## Data Availability

The original contributions presented in the study are included in the article/Supplementary Material, further inquiries can be directed to the corresponding author.
